# Dynamics of the Leaf-Litter Arthropod Fauna Following Fire in a Neotropical Woodland Savanna

**DOI:** 10.1371/journal.pone.0007762

**Published:** 2009-11-09

**Authors:** Heraldo L. Vasconcelos, Renata Pacheco, Raphael C. Silva, Pedro B. Vasconcelos, Cauê T. Lopes, Alan N. Costa, Emilio M. Bruna

**Affiliations:** 1 Instituto de Biologia, Universidade Federal de Uberlândia, Uberlândia, Minas Gerais, Brazil; 2 Department of Wildlife Ecology and Conservation and Center for Latin American Studies, University of Florida, Gainesville, Florida, United States of America; University of Lancaster, United Kingdom

## Abstract

Fire is an important agent of disturbance in tropical savannas, but relatively few studies have analyzed how soil-and-litter dwelling arthropods respond to fire disturbance despite the critical role these organisms play in nutrient cycling and other biogeochemical processes. Following the incursion of a fire into a woodland savanna ecological reserve in Central Brazil, we monitored the dynamics of litter-arthropod populations for nearly two years in one burned and one unburned area of the reserve. We also performed a reciprocal transplant experiment to determine the effects of fire and litter type on the dynamics of litter colonization by arthropods. Overall arthropod abundance, the abundance of individual taxa, the richness of taxonomic groups, and the species richness of individual taxa (Formiciade) were lower in the burned site. However, both the ordinal-level composition of the litter arthropod fauna and the species-level composition of the litter ant fauna were not dramatically different in the burned and unburned sites. There is evidence that seasonality of rainfall interacts with fire, as differences in arthropod abundance and diversity were more pronounced in the dry than in the wet season. For many taxa the differences in abundance between burned and unburned sites were maintained even when controlling for litter availability and quality. In contrast, differences in abundance for Collembola, Formicidae, and Thysanoptera were only detected in the unmanipulated samples, which had a lower amount of litter in the burned than in the unburned site throughout most of our study period. Together these results suggest that arthropod density declines in fire-disturbed areas as a result of direct mortality, diminished resources (i.e., reduced litter cover) and less favorable microclimate (i.e., increased litter desiccation due to reduction in tree cover). Although these effects were transitory, there is evidence that the increasingly prevalent fire return interval of only 1–2 years may jeopardize the long-term conservation of litter arthropod communities.

## Introduction

Fire is a common and important agent of disturbance in many temperate and tropical ecosystems [1]. In the largest and most threatened savanna biome of South America – the Cerrado – fire is considered a principal determinant of vegetation structure and diversity [2], [3]. The occurrence of fires in the Cerrado region of central Brazil is ancient, dating back to 32,000 years before present (YBP) [4]. Consequently, the presence of mechanisms promoting tolerance to fire – such as insulation of living internal tissues through suberization of trunks and branches – is relatively common in the local flora [5]. Lightning strikes and indigenous activities, especially hunting, were the main causes of fire in the Cerrado prior to the arrival of the European colonizers. In contrast, fire is now mostly caused by agricultural activities [2], [5]. Although relatively little is known about the natural frequency of fires in the Cerrado, it is clear that the intensification of human activities in this region, especially during recent decades, has increased the frequency and extent of fires well above historical levels. The occurrence of fires at intervals of 1 to 3 years is now common in many areas [5], [6]; such short fire-return intervals reduce the density of woody vegetation, especially through mortality of young individuals and the more fire-sensitive (forest-adapted) species [2], [7]. Consequently, recurrent fires tend to convert Cerrado physiognomies into more open savannas or grasslands, while fire exclusion favors the expansion of forests in areas of suitable soil fertility [7], [8].

It has been suggested that the altered frequency and intensity of fires is in the Cerrado is causing widespread change in this ecosystem [9]. While the effect of fire on vegetation is relatively well established, however, few studies have evaluated how fire influences the Cerrado's fauna [10]–[12]. Furthermore, to our knowledge none of these studies have focused on leaf-litter arthropods, despite the critical role these organisms play in nutrient cycling and other biogeochemical processes [13], [14]. Studies in tropical and temperate ecosystems indicate that the response of soil-and-litter dwelling arthropods to both prescribed and accidental fire is variable. Some studies, especially those in forest habitats, have indicated that the abundance of these animals is severely and negatively impacted by burning [e.g., 15], [16], while others showed only minor effects [e.g., 17], [18], [19]. The response of individual taxa can also be variable, and some taxa may even show increased abundances in burned sites as a consequence of changes in habitat structure and/or microclimate [17], [18]. Finally, while some studies show quick recovery for most taxa [18], [20], others indicate that changes are detectable up to several years following a fire [15], [16].

As in other tropical savannas, litter-dwelling arthropods in the Cerrado are more prevalent in more closed physiognomies [21] (i.e., in those with a higher density of woody vegetation) and therefore a relatively well developed litter layer. Fire in these physiognomies can detrimentally affect litter fauna directly by killing individuals unable to escape from fire. However, fire can also exert indirect effects on arthropod communities. For instance, fire can reduce the quantity of litter available for arthropods to colonize [22], [23]. It can also alter the composition of the leaf-litter layer, because surviving trees often drop all or most of their green leaves in response to intense heat [17]. However, our understanding of how these changes in leaf-litter structure influence the associated arthropod fauna are for the most part unknown. If the main effect of fire on litter arthropods is a direct one, then we would expect to detect differences in arthropod populations between burned and unburned areas even if the amount and composition of litter available to invertebrates is held constant. If fire's effects are predominantly indirect, however, then differences in arthropod populations between burned and unburned areas would only be expected when there are also differences in litter quantity and/or composition. To resolve among these alternatives, we conducted a series of observational and manipulative experiments in which we monitored arthropod populations in a burned woodland savanna site and an adjacent unburned site over the course of 22-months. Our study addressed the following three questions. First, how does fire influence the abundance and diversity of litter arthropods? Second, for how long after a fire do these differences persist? Third, are differences in arthropod populations driven by differences in litter quantity or litter composition? Our findings indicate that fire reduces overall arthropod abundance, the abundance of individual taxa, and the richness of taxonomic groups. For some taxa reduced abundance appears to be a consequence of the lower availability of litter in the fire affected areas, while for others direct mortality is the most likely explanation. Fire effects on leaf-litter arthropods of our Neotropical woodland savanna, however, were transitory and in most cases negligible in the second year after fire.

## Results

### Dynamics of the Arthropod Fauna Following Fire

Over the course of 22 months we collected 10,703 arthropods from 16 orders and one family in the 160 unmanipulated litter samples. With 3,779 individuals Formicidae was by far the most common taxon, followed by Acari (N = 1866) and Colembola (N = 1070). Among ants, there was a total of 68 species, representing 28 genera of which *Pheidole* was the most diverse, with a total of 12 species.

**Figure 1:**
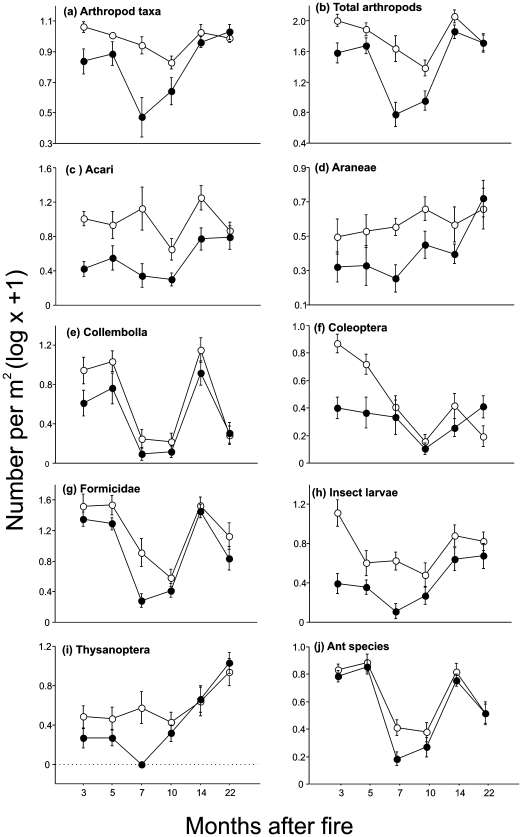
Dynamics of leaf-litter arthropods communities following a fire. (A) Number of arthropod taxa, (B) total abundance of arthropods, (C–I) abundance of individual taxa, and (J) species richness of ants in unmanipulated litter from burned (filled symbols) and unburned sites (open symbols) 3 to 22 months after the fire. Values represent mean numbers (±SE) per m^2^.

**Figure 2:**
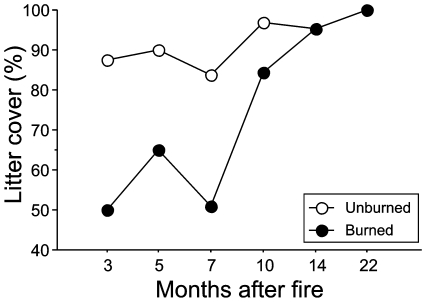
Effect of fire on leaf-litter cover. Percentage of litter cover in burned and unburned sites 3 to 22 months after the fire.

Samples from the burned site contained, on average, fewer arthropod taxa and fewer individuals (Fig. 1a and b). However, differences between burned and unburned sites in overall arthropod abundance and in the mean number of arthropod taxa per sample depended on time since fire (fire x time interaction, Table 1). During the first year differences in arthropod abundance and in the richness of arthropod taxa between burned and unburned sites were much greater during the dry season (7–10 months after the fire) than during the wet season (3–5 months after the fire). During the second year, when differences in litter cover between burned and unburned areas disappeared (Fig. 2), differences in overall arthropod abundance and in richness of arthropod taxa also tended to disappear (Fig. 1).

**Table 1:** Results of two-way ANOVAs evaluating the effects of fire and time since fire on the abundance and richness of arthropods in unmanipulated litter samples (*N* = 136)^a^.

	Fire		Time		Fire x Time	
Variable	*F_1,124_*	*P*	*F_5,124_*	*P*	*F_5,124_*	*P*
No. arthropod taxa	19.12	<**0.001**	9.04	<**0.001**	2.67	**0.025**
Total of arthropods	26.21	<**0.001**	19.69	<**0.001**	2.38	**0.043**
Acari	30.62	<**0.001**	4.81	<**0.001**	1.22	0.305
Araneae	9.30	**0.003**	2.40	**0.041**	0.64	0.667
Collembola	6.65	**0.011**	25.04	<**0.001**	0.56	0.732
Coleoptera (adults)	9.90	**0.002**	12.27	<**0.001**	4.21	**0.001**
Formicidae	12.27	**0.001**	28.96	<**0.001**	1.04	0.394
Insect larvae and pupae	25.21	<**0.001**	6.02	<**0.001**	1.86	0.107
Thysanoptera	4.90	**0.029**	7.61	<**0.001**	1.46	0.206
Species richness of ants	4.85	**0.029**	38.05	<**0.001**	0.97	0.438

a Significant effects (*P*<0.05) appear in bold.

Similar results were obtained when we analyzed the effects of fire on the species richness of ants, and on the individual abundances of the seven most common arthropod taxa. For each one of these seven taxa we found, on average, significantly fewer individuals in samples from the burned than from the unburned site (Table 1). Although a significant fire x time interaction was only detected for Coleoptera (Table 1), visual inspection of the graphs suggest that for most taxa differences in abundance between burned and unburned sites had dissipated by the second year (i.e., 14–22 months after the fire; Fig. 1). Similarly, differences in the species richness of ants (number of species per sample) between burned and unburned sites were negligible during the second year after fire (Fig. 1i).

**Figure 3:**
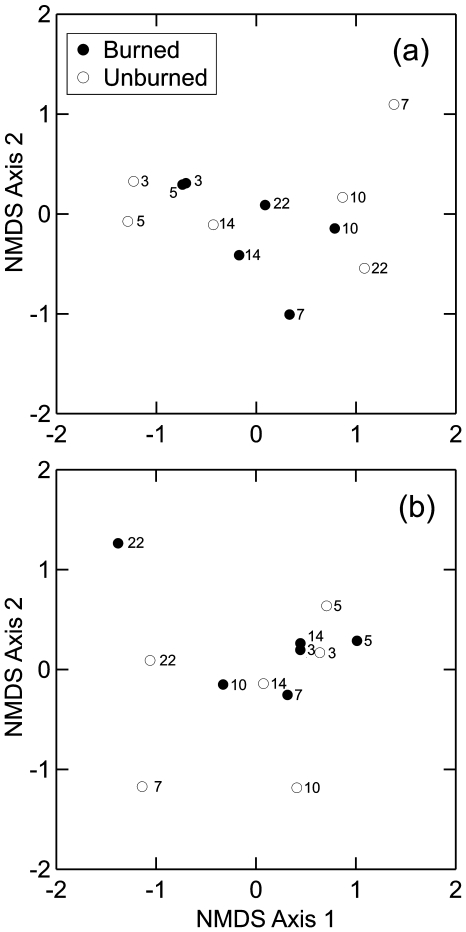
Effect of fire and time since fire on the composition of leaf-litter arthropod communities. (A) NMDS ordination of burned and unburned sites based on the ordinal-level composition of the litter arthropod fauna (relative abundances of different arthropod taxa). (B) NMDS ordination of burned and unburned sites based on the species composition of litter-dwelling ants (relative frequencies of different species). Number near symbols represent the number of months elapsed since the fire event.

NMDS ordination analysis did not reveal a separation between burned and unburned sites with regard to faunal composition of leaf-litter samples taken at different times since the fire event (Fig. 3). This probably reflects the fact that fire detrimentally affected the absolute abundances of all leaf-litter arthropod taxa. Consequently, their relative abundances did not change between the burned and unburned sites. Most of the observed differences in faunal composition seems to be attributed to rainfall seasonality, with samples taken during the dry season (regardless of their site of origin) being located at the left side of the ordination plot whereas those taken during the wet season were located at the right side (Fig. 3a). Seasonal changes in the composition of the leaf-litter fauna are probably explained by a decline in the relative abundance of ants (Formicidae) and springtails (Collembola) in samples taken during the dry season (7, 10, and 22 months after fire; Fig. 1e and g). Comparable results were obtained with regard to the species composition of the leaf-litter ant fauna. The latter showed a much stronger response to rainfall seasonality than to fire disturbance (Fig. 3b).

### Litter Transplant Experiment

Litter type did not significantly affect the abundance or richness of leaf-litter arthropods (Table 2). On average we found 48.7±6.4 (S.E.) individuals and 7.4±0.5 arthropod taxa per m^2^ in samples with “normal” litter and 51.8±5.6 individuals and 7.4±0.4 taxa in samples with “green” litter. Similarly, we found 4.06±0.44 and 4.14±0.44 species of ants in samples with normal and green litter, respectively.

**Table 2:** Results of three-way ANOVAs evaluating the effects of fire, litter type and time since litter transplant on the overall abundance of arthropods, richness of arthropod taxa, and abundance of individual taxa in experimental litter plots (*N* = 128)^a^.

	Fire		Litter type		Time	
Variable	*F_1,115_*	*P*	*F_1,115_*	*P*	*F_3,115_*	*P*
No. arthropod taxa	15.33	<**0.001**	0.09	0.768	11.83	<**0.001**
Total of arthropods	14.62	<**0.001**	0.62	0.433	35.71	<**0.001**
Acari	39.12	<**0.001**	0.95	0.331	2.09	0.106
Araneae	6.67	**0.011**	1.13	0.209	5.02	**0.003**
Collembola	0.49	0.486	1.45	0.231	64.02	<**0.001**
Coleoptera (adults)	4.89	**0.029**	0.51	0.476	18.42	<**0.001**
Formicidae	0.42	0.516	0.07	0.788	93.00	<**0.001**
Insect larvae and pupae	7.48	**0.007**	2.92	0.090	4.68	**0.004**
Thysanoptera	1.90	0.171	0.04	0.846	1.26	0.290
Species richness of ants	0.25	0.622	0.14	0.707	105.16	<**0.001**

a Significant effects (*P*<0.05) appear in bold.

None of the interactions between main factors were significant (results not shown).

**Figure 4:**
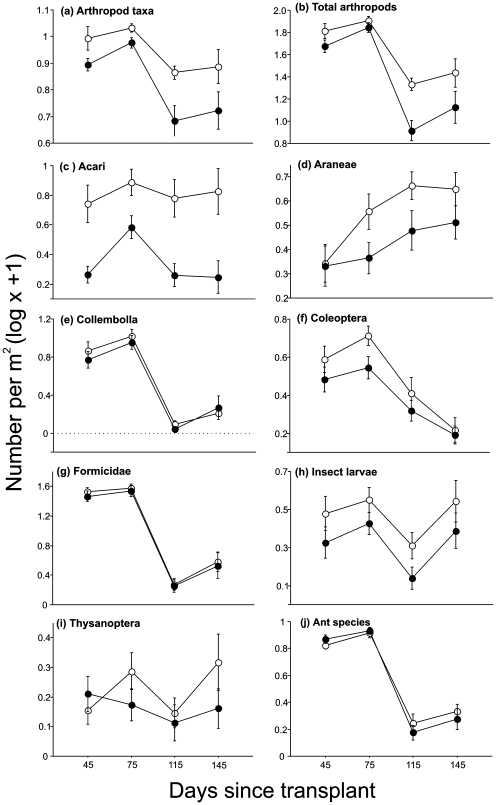
Abundance and diversity of arthropods in litter transplanted into burned and unburned sites. (A) Number of arthropod taxa, (B) total abundance of arthropods, (C–I) abundance of individual taxa, and (J) species richness of ants in litter transplanted into burned (filled symbols) and unburned sites (open symbols), 45 to 145 days after transplant. The litter transplant experiment started ca. 2.5 months post-fire. Values represent mean numbers (±SE) per m^2^. Data from different litter types were combined.

Litter transplanted into the burned site contained significantly fewer arthropod groups, less ant species, and fewer arthropod individuals than litter transplanted into the unburned site (Table 2; Fig. 4). However, the effect of fire on the abundance of arthropods colonizing experimental litter was idiosyncratic. While fire negatively affected the abundance of Acari, Araneae, adult Coleoptera, and insect larvae and pupae, it had no significant effect on Colembola, Formicidae, and Thysanoptera (Fig. 4, Table 2).

The amount of time that had elapsed since the litter transplant also affected the abundance and richness of arthropods in the experimental plots (Table 2). However, Araneae was the only taxon to show a monotonic increase in abundance through time (Fig. 4), indicating that only for this taxon was the colonization of new individuals continuous throughout the experiment. Two taxa – Acari and Thysanoptera – did not show any significant variation in abundance through time. Others only showed a response to seasonality in rainfall, with more individuals found in samples collected during the wet season (45–75 days after the transplant) than during the dry season (115–145 days after transplant; Fig. 4).

## Discussion

To our knowledge this is the first comprehensive study of the leaf-litter arthropod fauna of the Brazilian Cerrado and its response to disturbance by fire. Consequently, no data for other Cerrado sites with which to compare our results are available. Nevertheless, the ordinal-level composition of the litter arthropod fauna of the Cerrado site we studied appears similar to that of other tropical woodland savannas. For instance, ants, mites, springtails, spiders, and beetles also comprised most of the arthropod individuals collected in the woodland savannas of northern Australia [18].

Overall, our results indicate that fire detrimentally affected the litter arthropod fauna in multiple ways: it reduced overall arthropod abundance, the abundance of individual taxa, the richness of taxonomic groups, and the species richness of individual taxa (Formiciade). However, both the ordinal-level composition of the litter arthropod fauna and the species-level composition of the litter ant fauna were not dramatically altered by fire. In fact, most of the observed changes in the composition of these assemblages were related to rainfall seasonality and not to fire disturbance.

That for many taxa (Acari, Araneae, Coleoptera, Thysanoptera, and insect larvae and pupae) the differences in abundance between burned and unburned sites were maintained even when controlling for litter availability and quality suggests that these differences were driven by a direct effect of fire on arthropod populations rather than an indirect effect on their resources (litter). In contrast, differences in abundance for Collembola and Thysanoptera, and in abundance and species richness for Formicidae, were only detected in the unmanipulated samples, which had a lower amount of litter in the burned than in the unburned site throughout most of our study period. Here, the effect of fire may have simply been a consequence of the lower availability of litter in the fire affected areas. This is not to say that fire did not have any immediate and direct impact on Collembola, Formicidae, and Thysanoptera populations, only that if there was a direct effect of fire on these populations it was brief and we were unable to detect it. It is more likely that individuals from these groups escaped from fire by finding refuge in the lower soil horizons or other safe sites. Many Cerrado ants nest deep in the soil and therefore appear resistant to direct effects [24] as also observed in other tropical savannas [25], [26]. Similarly, springtails (Collembola) –which are highly sensitive to desiccation [27], and most likely migrate into the soil during the Cerrado dry season (Fig. 1) – may have similarly escaped from the direct effects of fire. Together these results indicate that, as observed in other fire-prone ecosystems [18], [22], arthropod responses to fire in the Cerrado vary among different taxonomic groups.

Nutrient reabsorption during leaf senescence is a common feature of Cerrado trees [28]. Consequently, nutrient content is lower in senescent leaves that fall from trees and are incorporated into the litter layer than in mature and young leaves that remain on trees [28]. Because intense heating caused trees from the burned area to suddenly drop their leaves, there may have been limited opportunity for trees to reabsorb nutrients present in them. Leaves in the litter from the burned area were also structurally distinct – they were relatively more intact and therefore occupied a greater volume than the partially consumed leaves in the litter from the unburned area (pers. obs.). Litter structure and nutrient content have both been found to affect the abundance and composition of the leaf-litter fauna in some systems [29], [30]. However, we did not detect a significant effect of litter type on arthropod abundance or diversity, despite the fact that litter produced in the burned area was structurally different and presumably had higher nutrient content than litter from the unburned area. Nevertheless, it is important to note that although abundances at the ordinal level were similar, there may have been species-level responses to differences in litter type.

Rainfall seasonality is a defining characteristic of the Cerrado biome region [31] with myriad consequences for this ecosystem. It is therefore not entirely surprising that we detected a strong effect of seasonality on the litter arthropod fauna. Overall, we found fewer individuals and fewer species or groups of arthropods in the litter samples taken during the dry than during the wet season – results similar to those observed in deciduous forests of Panama [32]. However, we were surprised to find apparently synergistic impacts of rainfall and fire on litter arthropod populations. Differences in overall arthropod abundance and in richness of arthropod groups between burned and unburned areas tended to disappear five months post-fire (Fig. 1). However, these differences became more pronounced with the onset of the dry season (7 months after the fire) than at any other period during the preceding wet season. Similarly, differences in ant species richness between burned and unburned sites were greatest during the first dry season after the fire event. One possible explanation for this pattern is that reduced tree cover in the burned area made litter particularly more prone to desiccation during the dry season. Litter water content is a key determinant of arthropod abundance and diversity in the seasonal tropics, and watering of litter during the dry season induces arthropod migration into wetter litter patches [33]. Because litter from the burned area was more exposed to sun it is reasonable to suppose that migration of arthropods into deeper and wetter soil horizons occurred more frequently in the burned than in the unburned area, especially during the drier periods of the year.

Differences in arthropod abundance, richness of taxonomic groups, and richness of species between the burned and unburned sites were for the most part negligible during the second year of our study, and this was true for samples taken during both the dry and wet seasons. This strongly suggests that the differences observed during the first year of our study were not due to a pre-existing difference between the burned and unburned sites, but rather due to the direct and indirect impacts of fire on the litter arthropod fauna. Our results thus indicate a nearly complete recovery of the litter arthropod community in less than two years after the fire, a result comparable to those of other similar studies [17], [19], [20], [but see 22]. Here, we attribute the recovery of the arthropod fauna to a concomitant recovery of the litter layer and plant cover. There was a luxuriant growth of small trees, shrubs, and herbs in the burned area about one year after the fire (pers. obs.). The formation of such a dense plant layer is likely to have diminished the intensity or even eliminated completely any microclimatic differences between burned and unburned areas.

It is important to note, however, that in our experimental design there was only one burned and one unburned site. This lack of site replication, which is typical in studies of large scale disturbances such as fires, hurricanes, and drought events [34]–[37], necessarily limits our inference to sites we studied. Indeed, it is even possible that the responses we observed are actually the result of inter-site differences not directly related to fire [38]. However, we are confident that this possibility is extremely unlikely, especially in the light of short-term nature of the observed responses. Furthermore, our substantial replication within site and our combination of experimental and observational approaches gives us confidence that the patterns we have documented are representative of the effects of fire in this Cerrado physiognomy. An important next step is to expand our study to other pairs of burned and unburned sites to evaluate the generality of our results.

In contrast to the savannas of Africa and Australia[39], [40], fire is rarely used as a management tool in Cerrado protected areas except those dominated by grasslands where fire prevention can result in catastrophic fires [41]. However, fire is used by Brazilian farmers on a nearly annual basis to manage their land [2]. Such fires very often penetrate into conservation areas; as a result, fire return intervals as short as one year have been recorded in some areas [6]. This is of particular concern for the conservation of many aspects of the Cerrado biota, including the leaf-litter fauna which – as indicated here – will not be able to survive an annual or bi-annual fire regime. Measures that minimize the occurrence of accidental fires in protected areas of the Cerrado are thus urgently needed, especially in light of predictions from climatic models that project a 3.5°C temperature increase across South America by 2080–2099 [42]. Such an increase could potentially increase the frequency and intensity of fire across the biome, exacerbating the negative effects of fire documented in our and other studies.

## Materials and Methods

### Study Site

**Figure 5:**
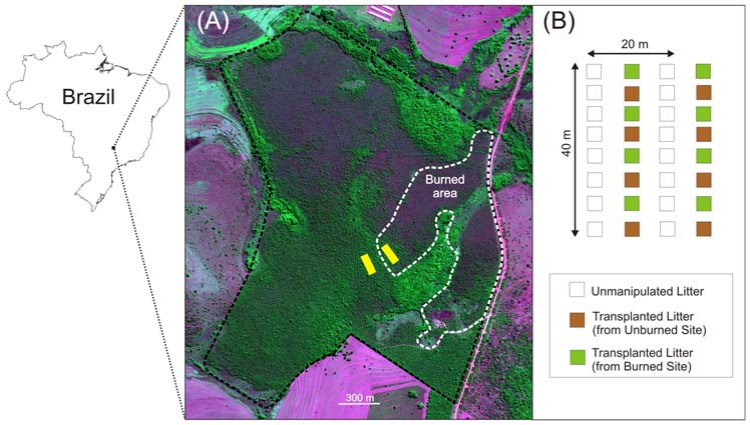
Image of the study area and layout of the sampling design. (A) Quickbird satellite image of the study site (*Estação Ecológica do Panga*, Minas Gerais, Brazil) taken approximately one year prior to the fire that was the focus of our studies. Rectangles represent the location of the transects in the burned and unburned study sites. (B) Schematic view of the distribution of the litter sampling plots along each 40-m long transect. In each site we established a total of 10 transects from which we collected unmanipulated litter samples at different times after fire and a total of eight transects into each we transplanted litter originated from the burned and unburned sites to plots of 1-m^2^.

The study was conducted at the Estação Ecológica do Panga (19°10′S, 48°23′W), a 404 ha reserve located 30 km south of Uberlândia, Minas Gerais, Brazil (Fig. 5). This reserve contains most of the major Cerrado plant physiognomies, and has been described as one of the best-preserved Cerrado sites in southeastern Brazil [43]. Our experiments were conducted in an area covered by *cerrado denso* [3], which is a savanna woodland with abundant shrubs and trees reaching 4–10 m in height and a sparse grass cover. The region is characterized by a subtropical climate with two well-defined seasons: a dry winter (April to September) and a rainy summer (October to March). The mean annual temperature and precipitation are 22°C and 1650 mm, respectively. Soils at the site are primarily red latosols that vary from moderately to strongly acidic.

**Figure 6:**
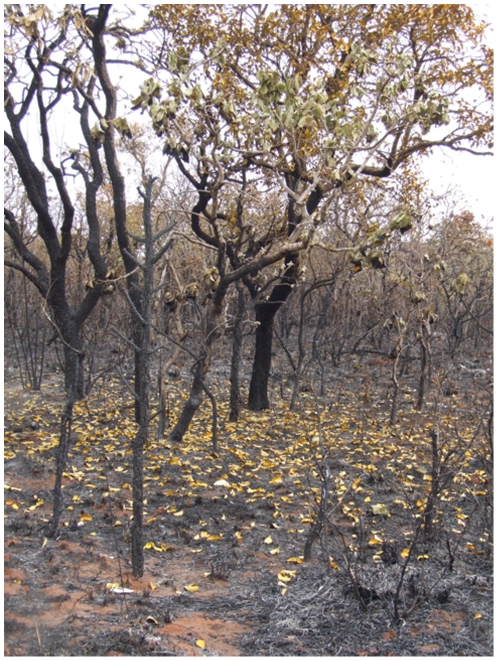
General view of the burned site three days after the fire. Note the leaves recently dropped by fire-affected trees (“green litter”).

Although Panga has no prescribed burn program, there are occasional fires due to human activities surrounding the reserve. Since its creation, in 1986, the reserve has been partially burned in 1992, 2003 and 2006. Our study took place following the 2006 fire, which occurred on September 15 of that year. This fire started at approximately 11 a.m. and was controlled by an anti-fire brigade within a few hours. Nevertheless, about 20% of the area of the reserve was burned (Fig. 5), with flames of 1–2 m consuming all vegetation to this height. Although the crowns of larger trees were not burned, these trees still dropped all or most of their leaves within a few weeks (Fig. 6). Most of the leaves dropped by fire-affected trees were relatively intact because the fire occurred during the period when trees were flushing new leaves. The litter produced by those trees was therefore qualitatively different from that in the unburned area, which was composed of partially-consumed senesced-leaves.

### Dynamics of the Arthropod Fauna Following Fire

We examined the dynamics of litter-arthropod populations for a period of nearly two years after the fire. To do so we established ten line-transects in a site within the fire-affected area and ten transects in a nearby part of the reserve that was structurally similar but not burned. Transects in the unburned site were located ca. 100 m away from the edge of the burned area (Fig. 5). Although there are reports of high temperatures and smoke affecting trees located up to 2 km from the burned area [44], this was not the case in our study area where trees about 30–40 meters or more from the edge of the fire-affected area remained intact (Vasconcelos, H.L., pers. obs.). A key advantage of our sampling and experimental design is that working in adjacent burned and unburned areas allows us to minimize variation in soil structure, topography, and other factors that could affect the abundance and diversity of litter-arthropods. This is of particular importance given the diversity of physiognomies at the study site; for these reasons we chose to work in adjacent burned and unburned portion of the same vegetation physiognomy (cerrado denso). The adjacent burned and unburned study sites have the same soil type (red latossol), and their vegetation structure was virtually identical prior to fire (Vasconcelos, H.L., pers. obs.; see also Fig. 5).

Transects were 40 m long and arranged parallel to each other, with a distance of 20 m between two adjacent transects (Fig. 5). Eight 1 m^2^ plots were set and evenly spaced along each transect. We collected the litter from all plots in randomly selected transects within each site 3, 5, 7, 10, 14 and 22 months after the fire; we collected from two transects at all sampling dates, except 5 and 22 months after the fire, when due to time constraints it was only possible to collect from one transect. Different transects were sampled at different dates, so that the same plot was never re-sampled. Prior to these collections we measured litter cover (i.e., litter present or absent) in the four corners of each plot. The litter removed from each plot was sieved through a 0.8 cm mesh; we then extracted arthropods from the sifted litter by placing litter in a Winkler extractor [45] for 48 h. These arthropods were identified to order or family (in the case of Formicidae only), with the exception of the immature insects which were grouped as “insect larvae and pupae”. Ants were further identified to species/morphospecies level. Voucher specimens are deposited at the Zoological Collection of the Universidade Federal de Uberlândia (UFU), in Uberlândia, Brazil. A complete list of the species/morphospecies is available from the first author upon request.

### Litter Transplant Experiment

For this experiment we established eight additional transects in each site ca. 2.5 months after the fire. We removed the litter existing from all plots of all transects and collected additional litter samples from areas just outside of the transects. Litter from the burned and unburned sites were kept separate. The litter from each site was thoroughly mixed and dried for 72 h at 60°C to kill the associated fauna. We then added 760 g (dry weight) of arthropod-free litter (600 g of leaves and 160 g of twigs <2 cm in diameter) to each plot. This amount of litter corresponds to the average litter biomass on the ground of the unburned site at the time of our study (leaves = 591.2±152.8 g, twigs = 166.4±89.2 g, mean±SD). Half of the plots within each transect received only litter collected in the unburned site, while the other half received only the litter from the burned site (Fig. 5). The transplanted litter was collected for extraction of the associated arthropods at 45, 75, 115 and 145 days after the transplant (i.e., ca. 4, 5, 6 and 7 months post-fire). On each occasion two transects were randomly selected for sampling.

We evaluated the effects of site (burned vs. unburned), litter type (senesced litter from unburned site vs “green” litter from the burned site) and time since litter transplant (4, 5, 6 and 7 months post-fire) on the overall abundance of arthropods, the richness of arthropod groups, and the richness of ant species in our experimental plots using a three-way factorial ANOVA. Similarly, a two-way factorial ANOVA was used to evaluate the effects of fire and time of sampling (3, 5, 7, 10, 14 and 22 months after the fire) on the overall abundance of arthropods, on the richness of arthropod groups, and on the richness of ant species in the unmanipulated litter plots. Data were log (x+1) transformed in order to meet the assumptions of parametric statistics.

We used Non-Metric Multidimensional Scaling (NMDS) to ordinate samples taken at different times in the burned and unburned sites by their faunal similarity. Faunal similarity was estimated using the Bray-Curtis index, based on the relative abundances of each arthropod group at each site in a given sampling date. Similarly, we used NMDS to ordinate samples based on the similarity of their ant assemblages. Similarity in ant species composition (Bray-Curtis index) was based on the relative frequencies of each ant species at each site in a given sampling date. All statistical analyses were done using the default options of Systat 10 [46].
